# Multicenter Evaluation of Antibiotic Use and Antibiotic Stewardship Programs in Latin American Hospitals

**DOI:** 10.1093/ofid/ofaf364

**Published:** 2025-06-25

**Authors:** Valeria Fabre, Sara E Cosgrove, Yea-Jen Hsu, Twisha S Patel, Fernanda C Lessa, Andrea Alvarado, Bowen Aquiles, Ana B Arauz, Maria F Barberis, Maria Del Carmen Bangher, Maria P Bernachea, Marisa L Bernan, Alfredo Canton, Ximena Castañeda, Angel M Colque, Rosa Contreras, Wanda Cornistein, Silvia Mabel Correa, Gustavo Costilla Campero, Marta Isabel Chamorro Ayala, Lidia Espinola, Clara Esquivel, Cecilia Ezcurra, Johana Fernandez, Sandra Ferrari, Natalia Frassone, Carlos Garcia Cruz, Maria Isabel Garzón, Carlos H Gomez Quintero, José A Gonzalez, Lucrecia Guaymas, Fausto Guerrero-Toapanta, Sandra Lambert, Diego Laplume, Itzel L Lopez, Herberth Maldonado, Noelia Mañez, Diego M Maurizi, Mario Melgar, Florencia Mesplet, Carlos Morales Pertuz, Cristina Moreno Izquierdo, Luciana Gabriela Moya, Yanina Nuccetelli, Glendys Núñez, Argelis Olmedo, Belén Palacio, Antonella Pauluzzi, Mariana de Paz Sierra, Florencia Pellice, Loraine Perez Alvear, Carla Lorena Raffo, Fanny Reino, Ligia Vence Reyes, Gerardo Ricoy, Viviana E Rodriguez, Federico Romero, Juan J Romero, Mariquena Ruiz, Maria Eugenia Russo, Graciela Sadino, Nancy Sandoval, Natalia Staffolani, Maria Jose Torralvo, Alejandra M Urueña, Hugo Videla, Marisol Valle, Silvia Vera Amate Perez, Hernan Vergara-Samur, Silvina Villamandos, Olmedo Villarreal, Eduardo Warley, Guadalupe Reyes-Morales, Rodolfo E Quiros

**Affiliations:** Division of Infectious Diseases, Department of Medicine, School of Medicine, Johns Hopkins University, Baltimore, Maryland, USA; Division of Infectious Diseases, Department of Medicine, School of Medicine, Johns Hopkins University, Baltimore, Maryland, USA; Department of Health Policy and Management, Johns Hopkins Bloomberg of School of Public Health, Baltimore, Maryland, USA; International Infection Control Branch, Division of Healthcare Quality Promotion, Centers for Disease Control and Prevention, Atlanta, Georgia, USA; International Infection Control Branch, Division of Healthcare Quality Promotion, Centers for Disease Control and Prevention, Atlanta, Georgia, USA; Unidad de Cirugia Cardiovascular de Guatemala, Guatemala, Guatemala; Hospital Sociedad de Lucha Contra el Cáncer, Guayaquil, Ecuador; Departamento de Medicina, Universidad de Panamá, Panama; Hospital Santo Tomas, Panama, Panama; Hospital Nacional Profesor Alejandro Posadas, El Palomar, Argentina; Instituto de Cardiología de Corrientes “Juana Francisca Cabral,” Corrientes, Argentina; Clínica Conciencia, Neuquén, Argentina; Hospital Interzonal General de Agudos San Roque, Buenos Aires, Argentina; Hospital Punta Pacífica Salud, Panamá, Panama; Clínica De La Mujer, Bogotá, Colombia; Hospital Mederi, Bogota, Colombia; Complejo Médico Churruca Visca, Buenos Aires, Argentina; Hospital Dr Marcial V. Quiroga, San Juan, Argentina; Hospital Universitario Austral, Buenos Aires, Argentina; Hospital Municipal de Trauma Dr Federico Abete, Malvinas Argentinas, Argentina; Hospital Angel C. Padilla, Tucumán, Argentina; Hospital Zonal General de Agudos Dr Alberto Eurnekian, Buenos Aires, Argentina; Hospital El Cruce, Buenos Aires, Argentina; Hospital San Benito, Peten, Guatemala; Hospital Alemán, Buenos Aires, Argentina; Hospital Dr Guillermo Rawson, San Juan, Argentina; Hospital Dr Guillermo Rawson, San Juan, Argentina; Clínica Universitaria Privada Reina Fabiola, Córdoba, Argentina; Hospital Sociedad de Lucha Contra el Cáncer, Guayaquil, Ecuador; Hospital Privado Universitario de Córdoba, Córdoba, Argentina; Clínica De La Mujer, Bogotá, Colombia; Hospital Militar Central, Bogotá, Colombia; Hospital Irma de Lourdes Tzanetatos, Panama, Panama; Clínica Provincial de Merlo, Buenos Aires, Argentina; Hospital Carlos Andrade Marín, Quito, Ecuador; Hospital El Cruce, Buenos Aires, Argentina; Hospital Nacional Profesor Alejandro Posadas, El Palomar, Argentina; Clínica Hospital San Fernando, Panama, Panama; Unidad de Cirugia Cardiovascular de Guatemala, Guatemala, Guatemala; Universidad del Valle de Guatemala, Guatemala, Guatemala; Hospital Italiano de Buenos Aires, Buenos Aires, Argentina; Hospital Municipal de Agudos Dr Leonidas Lucero, Bahía Blanca, Argentina; Hospital Roosevelt, Guatemala, Guatemala; Hospital Cesar Milstein, Buenos Aires, Argentina; Hospital Del Tunal, Bogotá, Colombia; Hospital Metropolitano, Quito, Ecuador; Clínica Conciencia, Neuquén, Argentina; Instituto de Diagnostico, La Plata, Argentina; Hospital Santo Tomas, Panama, Panama; Hospital Punta Pacífica Salud, Panamá, Panama; Sanatorio Allende Nueva Córdoba, Córdoba, Argentina; Hospital Universitario Austral, Buenos Aires, Argentina; Hospital Italiano de Buenos Aires, Buenos Aires, Argentina; Hospital Dr Marcial V. Quiroga, San Juan, Argentina; Hospital Del Tunal, Bogotá, Colombia; Hospital Municipal de Trauma Dr Federico Abete, Malvinas Argentinas, Argentina; Hospital Carlos Andrade Marín, Quito, Ecuador; The Panama Clinic, Panama, Panama; Complejo Médico Churruca Visca, Buenos Aires, Argentina; Hospital Alemán, Buenos Aires, Argentina; Sanatorio Allende Nueva Córdoba, Córdoba, Argentina; Hospital Vozandes, Quito, Ecuador; Hospital Cesar Milstein, Buenos Aires, Argentina; Hospital Interzonal General de Agudos San Roque, Buenos Aires, Argentina; Clínica Universitaria Privada Reina Fabiola, Córdoba, Argentina; Hospital Roosevelt, Guatemala, Guatemala; Maternidad Nuestra Señora De Las Mercedes De Tucumán, Tucumán, Argentina; Hospital Mederi, Bogota, Colombia; Maternidad Nuestra Señora De Las Mercedes De Tucumán, Tucumán, Argentina; Instituto de Diagnostico, La Plata, Argentina; Hospital Municipal de Agudos Dr Leonidas Lucero, Bahía Blanca, Argentina; Hospital Angel C. Padilla, Tucumán, Argentina; Hospital Militar Central, Bogotá, Colombia; Instituto de Cardiología de Corrientes “Juana Francisca Cabral,” Corrientes, Argentina; Hospital Irma de Lourdes Tzanetatos, Panama, Panama; Clínica Provincial de Merlo, Buenos Aires, Argentina; Division of Infectious Diseases, Department of Medicine, School of Medicine, Johns Hopkins University, Baltimore, Maryland, USA; Sanatorio Las Lomas, Buenos Aires, Argentina

**Keywords:** antibiotic use, implementation, Latin America, resources, stewardship

## Abstract

**Background:**

There is limited knowledge on the extent of antimicrobial stewardship program (ASP) implementation in health care facilities (HCFs) in Latin America.

**Methods:**

We performed an evaluation of ASPs in Latin American HCFs from March 2022 to February 2023 using a structured self-assessment survey associated with a scoring system that evaluated leadership support and accountability, resources, antibiotic stewardship actions, education, and antibiotic use (AU) monitoring and reporting. Additionally, we collected monthly AU data (antibiotic consumption and point prevalence surveys) and number of multidrug-resistant infections in medical-surgical intensive care units. Self-assessment scores were correlated with AU through multivariable regression models adjusting for bed size, country of HCF, and incidence of infections (when appropriate).

**Results:**

Of the 39 HCFs recruited for the study, all completed the self-assessment, 36 performed the point prevalence survey, and 29 collected antibiotic consumption data. The overall median self-assessment score was 252.5 (IQR, 212.5–285) for a maximum possible score of 335. A high self-assessment score (top quartile) was associated with higher guideline-compliant AU (odds ratio [OR], 8.63; 95% CI, 3.03–24.6; *P* < .001), higher use of directed therapy (OR, 2.11; 95% CI, 1.41–3.1; *P* < .001), and less consumption of anti–methicillin-resistant *Staphylococcus aureus* agents (OR, −8.59; SE = 4.12; *P* = .037) after adjusting for bed size, country, and incidence of methicillin-resistant *S aureus* infections.

**Conclusions:**

Higher-level ASP implementation in Latin American HCFs correlated with better compliance with AU guidelines and decreased the use of vancomycin in the intensive care unit, supporting the need to improve resources for ASPs.

## Teaser

The level of ASP implementation in Latin American healthcare facilities was associated with more optimal antibiotic use patterns in intensive care units emphasizing the critical need to support growth of this programs to combat antimicrobial resistance.

Antimicrobial-resistant infections are a growing threat to human health globally [1]. Recent forecasts estimate that Latin America, the Caribbean, and South Asia may carry the highest antimicrobial resistance (AMR) mortality rates in 2050 [2]. Antimicrobial stewardship programs (ASPs) can be highly effective in reducing AMR [3, 4]; therefore, they have become a major pillar of national action plans to combat AMR in health care settings. According to a recent survey, most Latin American countries are in the process of developing or implementing national AMR action plans [5]. Previous studies evaluating implementation of ASPs in hospitals in the region reported important barriers to effective and sustainable ASPs, such as a lack of protected time for the ASP team, inadequate access to infectious disease pharmacists, a lack of a safety culture, and suboptimal information and technology resources [6–8]. Many of these barriers also hinder development of robust infection prevention and control (IPC) programs in the region [9]. This is particularly important in Latin America where hospital-acquired infections remain a leading cause of antibiotic use (AU) [10].

To improve our understanding of the current state of ASP implementation in Latin American hospitals, we conducted (1) a multicenter evaluation including a cross-sectional self-assessment based on ASP core elements and (2) evaluations of AU in participating hospitals’ intensive care units (ICUs) including antibiotic consumption and indicators of AU. Our findings will help prioritize actions to strengthen ASPs in the region.

## METHODS

### Study Design and Setting

We evaluated ASPs in acute care health care facilities (HCFs) from Guatemala, Panama, Ecuador, Colombia, and Argentina between March 2022 and February 2023, using a multimodal approach consisting of (1) a cross-sectional self-assessment to assess the HCF ASP structure, resources, and antibiotic stewardship (AS) activities and (2) an evaluation of AU in the medical-surgical ICUs of participating HCFs including antibiotic consumption and point prevalence surveys of AU. Due to prior experience regarding access and availability of data such as antibiotic consumption and infection rates, we focused on the ICUs. HCFs were recruited through PROAnet, a regional research network previously used for other multicenter studies [6, 11]. The presence of an individual responsible for AS was required to participate. Country selection was discussed with officers from the Centers for Disease Control and Prevention (CDC), and HCF selection was discussed with national health authorities to ensure that study activities were not conflicting with ongoing activities related to AS in the country or region. Recruitment and study activities were coordinated by the Johns Hopkins University study team.

The study was approved by the Johns Hopkins Medical Institutional Review Board as nonhuman subjects research/quality improvement and as exempt by local institutional review boards. This activity was reviewed by the CDC, deemed not research, and was conducted consistent with applicable federal law and CDC policy (see, eg, 45 CFR part 46.102[l][2], 21 CFR part 56; 42 USC §241[d]; 5 USC §552a; 44 USC §3501 et seq).

### HCF ASP Self-assessment Tool

A multidisciplinary group of physicians, pharmacists, microbiologists, and implementation scientists with expertise in AS from different countries and regions, such as the United States, Latin America, Africa, and Southeast Asia, was convened to develop an evaluation tool to assess ASP implementation. The self-assessed evaluation tool was based on the CDC's “Core Elements of Human Antibiotic Stewardship Programs in Resource-Limited Settings” and the World Health Organization's (WHO’s) “Antimicrobial Stewardship Programmes in Health-Care Facilities in Low- and Middle-Income Countries: A WHO Practical Toolkit” [12]. The group used a determinants framework to ensure that a broad set of variables relevant to different settings was considered for survey development [13, 14]. The rigorous development process comprised multiple rounds of review and revision by the multidisciplinary expert team, an assembly of an external expert consensus panel using a Delphi-like approach, and international pilot testing. The final self-assessment for inpatient HCFs, the Global Antibiotic Stewardship Evaluation Tool (G-ASET), included 68 items organized into 5 domains [15]. An individual question could score from 0 to 5 points: for most questions, 5 points were given for an element “implemented,” 2.5 points for “partially implemented,” or 0 points for “not implemented”; for other questions, points were determined by how many answer choice options were selected. The domains and possible maximum score per domain were as follows: leadership, commitment, and accountability (maximum score, 65); resources, education, and training (65); antibiotic stewardship actions (25); antibiotic use tracking (105); monitoring and reporting (75). Overall and by domain, scores were converted to a percentage for further analysis: (score obtained/possible score) × 100. The G-ASET was shared electronically with the HCF AS contact in March 2023. Instructions on how to complete the G-ASET were included in the tool. HCFs were encouraged to involve the AS team and other relevant departments (rather than a single individual) to complete the G-ASET. HCFs were given 3 months to complete the G-ASET.

### AU Data

Antibiotic consumption was measured in defined daily doses per 100 patient-days per month for the adult medical-surgical ICU of participating HCFs. We used the WHO Anatomical Therapeutic Chemical classification to define systemic antibacterials (enteral and intravenous [IV]) [16]. Data were collected by the local AS team using a structured template and entered in a secure platform [17]. For analysis, antibiotic groupings included the National Healthcare Safety Network classification (adult broad-spectrum antibacterial agents predominantly used for hospital-onset infections, adult broad-spectrum antibacterial agents predominantly used for community-acquired infections, adult antibacterial agents predominantly used for resistant gram-positive infections, adult narrow-spectrum β-lactam agents, adult antibacterial agents predominantly used for extensively antibiotic-resistant bacteria [Supplementary Table 1]) [18], the AWaRe classification system [16], and other criteria established by the study team (Supplementary Table 2).

Local AS teams performed monthly point prevalence surveys of AU in the same medical-surgical ICUs where antibiotic consumption data were collected, as previously described [10]. Briefly, on the day of the survey, patients in the ICU were evaluated to determine if they were receiving systemic antibiotics. For those prescribed antibiotics, antibiotic data were registered via a structured electronic form into a secure platform as follows: what antibiotic, the indication for use, whether the indication was guideline compliant (per national and/or international guidelines if local guidelines did not exist), if microbiologic cultures were ordered, if therapeutic drug monitoring was performed (when appropriate), whether the antibiotic had been a de-escalation or escalation of initial treatment, and whether the antibiotic was renally dosed (if appropriate), [17]. For a list of AU indicators and how they were calculated, see Supplementary Table 2.

### Infections due to Multidrug-Resistant Organisms

The number of new infections due to methicillin-resistant *Staphylococcus aureus* (MRSA), extended-spectrum β-lactamase–producing Enterobacterales, and carbapenem-resistant Enterobacterales detected in patients after 48 hours of admission to the medical-surgical ICU and the number of patient-days were collected by local AS teams on a monthly basis following previously published recommendations [19]. Local AS teams were trained by the study team on data collection and case adjudication to improve consistency across sites. Additional information on case definition is described in Supplementary Table 3.

### Statistical Analysis

All data were analyzed by the study team. G-ASET responses were analyzed by descriptive statistics, and comparisons of responses between for-profit and nonprofit HCFs were analyzed with the χ^2^ test. To understand the association between G-ASET scores and AU, scores were analyzed as a continuous variable and a categorical variable (ie, the top-scoring one-third vs the two-thirds with lower scores). Antibiotic consumption and multidrug-resistant organism infections were treated as continuous variables. We constructed a correlation matrix using Spearman correlation coefficients to assess bivariate relationships across G-ASET domains and antibiotic consumption. We then built multivariate linear regression models with random intercepts to account for multiple antibiotic consumption data points from the same HCF. The models adjusted for the corresponding multidrug-resistant organism infection rates (eg, the incidence of MRSA when evaluating the association between the HCF G-ASET score and its ICU consumption of anti-MRSA agents). We also tested interaction terms of infection rate and high/low-score group to investigate whether infection rates modified the association.

To evaluate other aspects of AU relevant to ASPs (eg, guideline compliance, de-escalation), we examined the association between G-ASET scores and AU indicators using a generalized linear model with binomial family and logit link. Statistical analyses were performed in Stata version 18.0 (StataCorp LLC). Statistical significance was defined as *P* < .05.

## RESULTS

### ASP Implementation According to G-ASET Results

#### ASP Structure, Support, and Resources

**Figure 1.**
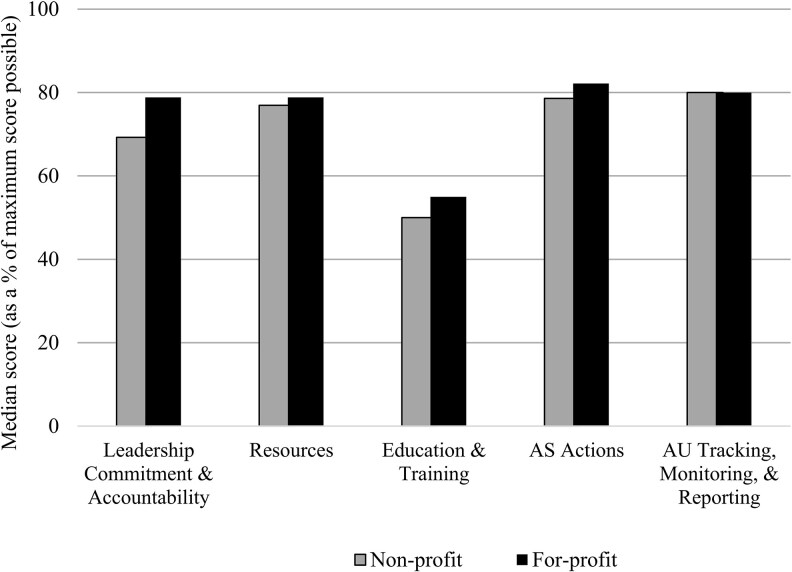
Median scores on the Global Antibiotic Stewardship Evaluation Tool among nonprofit (n = 22) and for-profit (n = 19) health care facilities in Latin American countries, March 2022–February 2023. No statistically significant differences were detected in any of the tool domains. AS, antibiotic stewardship; AU, antibiotic use.

Thirty-nine HCFs completed the G-ASET: 17 for-profit and 22 nonprofit. The HCFs had a median bed size of 183 (IQR, 120–286) and were from Argentina (n = 22, 56.4%), Colombia (n = 4, 10.3%), Ecuador (n = 4, 10.3%), Guatemala (n = 4, 10.3%), and Panama (n = 5, 12.8%). Except for the domain that assessed education, all others achieved a median score that corresponded to 70% of the maximum (Figure 1, Supplementary Table 4).

Among HCFs, 65% (for-profit) and 32% (nonprofit) indicated that AS was a priority (*P* = .04). Overall, 48% of HCFs had an ASP in their annual plan (70% for-profit vs 32% nonprofit, *P* = .04), and 64% indicated that there was a physician, pharmacist, and microbiologist collaborating on AS activities. A lack of any protected time for an ASP physician or pharmacist role was reported by 38% and 80% of HCFs, respectively. Most HCFs (85%) had an AS committee that met on a regular basis, although it had the authority to make decisions in only half of these HCFs. Most microbiology laboratories (85%) in participating HCFs were accredited, and 59% had implemented rapid diagnostic testing for infectious diseases.

#### Education

Forty-six percent of HCFs provided training opportunities to the AS team: 36% trained health care workers on AS topics during induction and 28% after hiring. Education to patients and families on AS-relevant topics was reported by 20% of HCFs.

#### AS Activities and Outcome/Process Measures

**Figure 2.**
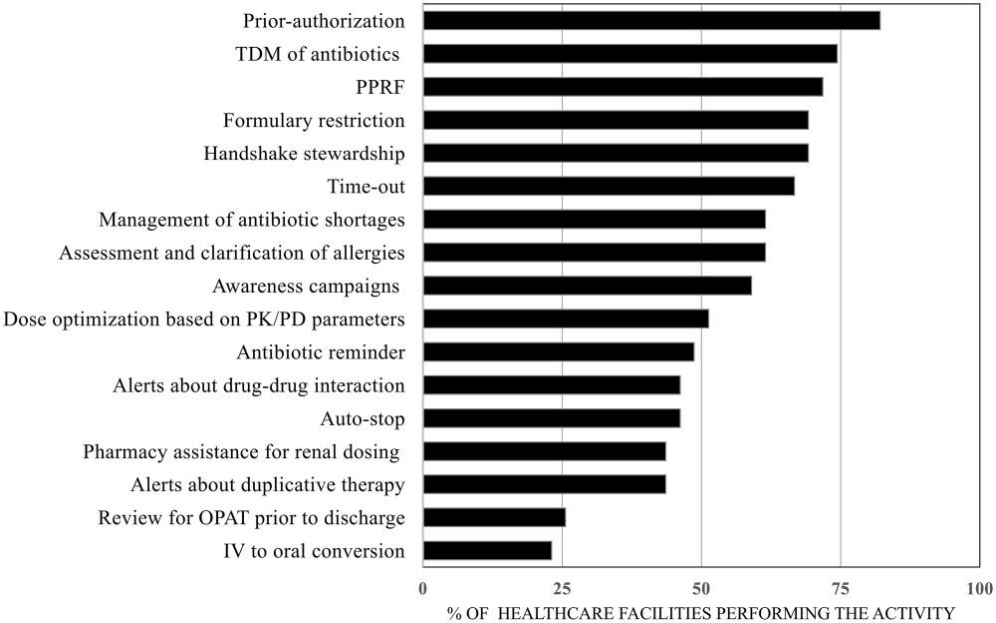
Antibiotic stewardship actions reported from the Global Antibiotic Stewardship Evaluation Tool by 39 health care facilities in Latin America, March 2022–February 2023. IV, intravenous; OPAT, outpatient parenteral antibiotics; PD, pharmacodynamic; PK, pharmacokinetic; PPRF, postprescription review with feedback; TDM, therapeutic drug monitoring.

Most HCFs (n = 37, 94.8%) had internal treatment guidelines for common infectious syndromes (eg, community-acquired pneumonia or intra-abdominal infections); however, 41% [16] stated that their guidelines included information regarding empiric antibiotic choice, recommended dose, duration of treatment, and alternative agents for patients with penicillin allergy. Additional AS strategies implemented by participating HCFs are shown in Figure 2. Prior authorization was the most common (n = 35, 89.7%) and occurred throughout the workday in 64.1% of HCFs [20]. Other common AS interventions included postprescription review with feedback (71.8%), discussion of antibiotics during unit rounds (69.2%), and formulary restriction (69.2%). Pharmacy-driven interventions, such as dose adjustment based on renal function, alerts about duplicative therapy, and IV-to-oral conversion, were less common (n = 17 [43.6%], n = 17 [43.6%], n = 9 [23.1%], respectively).

Eighteen (46.1%) HCFs reported that prior authorization was implemented in all units, while postprescription review with feedback was cited as a facility-wide intervention by 30.8% [12]. Supplementary Table 4 provides more details about whether AS interventions were implemented hospital-wide or for certain areas.

Fifty-one percent of HCFs developed and updated a cumulative antibiogram on a regular basis, and 56% reported updating treatment guidelines based on susceptibility patterns on the HCF antibiogram. More than 80% of HCFs reported measuring antibiotic consumption and antibiotic appropriateness. At least 1 of these AS metrics was reported to the HCF leadership and shared with prescribers by 41% and 30% of HCFs, respectively. Half of HCFs implemented quality improvement interventions based on AU data.

### Association of the Level of ASP Implementation With AU in Adult Medical-Surgical ICUs

Twenty-nine HCFs collected antibiotic consumption data for their adult ICUs. The median overall antibiotic consumption was 114 defined daily doses per 100 patient-days (IQR, 94–144). There were no significant differences in overall antibiotic consumption among HCFs based on bed size, type of ownership, country of origin, or G-ASET score (Supplementary Tables 5 and 6). However, when looking at specific antibiotic groups, we found that HCFs with a high score on the G-ASET (ie, top quartile) had significantly less ICU consumption of anti-MRSA antibiotics than HCFs with a low score (coefficient, −8.59; SE = 4.12; *P* = .037), after adjusting for incidence of MRSA infections in the unit, HCF bed size, and country (Table 1). There was also a trend toward lower consumption of carbapenems and the extensively antibiotic-resistant bacteria antibiotics among high-scoring HCFs compared with low-scoring HCFs after adjusting for extended-spectrum β-lactamase and carbapenem-resistant Enterobacterales infection rates in the unit, respectively (not statistically significant). Additionally, there was a statistically significant association between extended-spectrum β-lactamase and carbapenem-resistant Enterobacterales infection rates with consumption of carbapenems and extensively antibiotic-resistant bacteria antibiotics, respectively.

**Table 1.** Association of G-ASET Scores With Antibiotic Consumption (Dependent Variable) in Intensive Care Units (n = 29), March 2022–February 2023

	Daily Defined Dose per 100 Patient-Days								
	Anti-MRSA Antibiotics			Carbapenems			Extensively Antibiotic-Resistant Bacteria Antibiotics^a^		
	Coef	SE	*P* value	Coef	SE	*P* value	Coef	SE	*P* value
G-ASET score									
Low	Ref			Ref			Ref		
High^b^	−8.59	4.12	.037	−5.63	4.55	.216	−1.66	4.78	.728
Infection rate^c^	0.03	0.43	.936	0.65	0.17	<.001	0.80	0.18	<.001
Bed size									
≤200	Ref			Ref			Ref		
>201	2.02	3.75	.591	3.99	4.14	.335	−2.64	4.34	.543
Country									
Argentina	Ref			Ref			Ref		
Ecuador	−0.98	6.01	.870	8.31	6.64	.211	−7.80	6.96	.262
Guatemala	1.97	11.24	.861	16.64	12.44	.181	44.37	12.93	.001
Panama	26.18	6.13	<.001	32.40	6.75	<.001	20.54	7.09	.004

Multivariate linear regression models with random intercepts were used to account for monthly data from the same hospital.

Abbreviations: Coef, coefficient; G-ASET, Global Antibiotic Stewardship Evaluation Tool; MRSA, methicillin-resistant *Staphylococcus aureus*; Ref, reference.

^a^Extensively antibiotic-resistant bacteria antibiotics include the following agents: ceftazidime/avibactam, ceftolozane/tazobactam, colistimethate (intravenous only), polymyxin B (intravenous only), tigecycline.

^b^High score refers to G-ASET scores in the top quartile.

^c^Infection rate refers to the incidence of selected multidrug-resistant organisms in the intensive care units for which antibiotic consumption data were collected. For anti-MRSA infections, we controlled for the incidence of MRSA infections; for carbapenems use, we controlled for the incidence of extended-spectrum β-lactamase infections; for extensively antibiotic-resistant bacteria antibiotics, we adjusted for carbapenem-resistant infections.

The association between G-ASET scores and AU indicators was examined among 36 HCFs that performed point prevalence surveys (Table 2, Supplementary Table 7). HCFs with a high G-ASET score had higher use of antibiotics for directed therapy (odds ratio [OR], 2.11; 95% CI, 1.41–3.17; *P* < .001), higher guideline-compliant AU (OR, 8.63; 95% CI, 3.03–24.60; *P* < .001), and higher use of renally dosed antibiotics (OR, 2.83; 95% CI, 1.23–6.51; *P* = .014). There was a weak positive correlation between a high G-ASET score and higher use of anti-MRSA antibiotics prescribed for directed therapy in patients with a documented MRSA infection (OR, 3.23; 95% CI, .96–10.88; *P* = .059). There was no correlation between a high G-ASET score and the proportion of IV vancomycin undergoing therapeutic drug monitoring.

**Table 2.** Association of G-ASET Scores With Antibiotic Use Indicators (Dependent Variable) Derived From Point Prevalence Surveys Conducted in Intensive Care Units (n = 36), March 2022–February 2023

	Directed Therapy			De-escalation of Therapy			Dose Adjusted for Renal Function			Guideline Compliant Prescription			Targeted Therapy With Anti-MRSA Agent and MRSA Infection			Intravenous Vancomycin and TDM		
	OR	95% CI	*P* Value	OR	95% CI	*P* Value	OR	95% CI	*P* Value	OR	95% CI	*P* Value	OR	95% CI	*P* Value	OR	95% CI	*P* Value
G-ASET																		
Low	1			1			1			1			1			1		
High^a^	2.11	1.41–3.17	**<.001**	0.84	.34–2.10	.709	2.83	1.23–6.51	**.014**	8.63	3.03–24.60	**<.001**	3.23	.96–1.88	.059	2.81	.52–15.10	.227
Bed size																		
≤200	1			1			1			1			1			1		
>201	0.75	.51–1.10	.141	1.00	.53–1.88	.997	0.49	.24–.98	.042	1.24	.58–2.64	.579	1.07	.36–3.18	.904	0.21	.05–.87	.032
Country																		
Argentina	1			1			1			1			1			1		
Colombia	0.40	.26–.60	<.001	0.47	.14–1.58	.220	0.49	.14–1.71	.262	0.16	.03–.75	.020	0.53	.10–2.86	.460	0.00	.00–.00	<.001
Ecuador	0.60	.23–1.57	.299	0.11	.03–.33	<.001	1.82	.68–4.89	.233	17.38	3.34–9.52	.001	1.60	.31–8.23	.574	0.02	.00–.37	.008
Guatemala	0.72	.29–1.81	.488	0.00	.00–.00	<.001	0.48	.24–.93	.030	0.03	.01–.10	<.001	1.60	.37–6.96	.532	0.00	.00–.00	<.001
Panama	1.42	.74–2.73	.291	1.15	.41–3.22	.786	0.58	.16–2.14	.416	0.18	.08–.43	<.001	0.02	.00–.13	<.001	0.72	.11–4.82	.736

The multivariate regression model accounted for bed size and country of participating hospital. Bold values indicate statistically significant.

Abbreviations: G-ASET, Global Antibiotic Stewardship Evaluation Tool; MRSA, methicillin-resistant *Staphylococcus aureus*; TDM, therapeutic drug monitoring.

^a^High score refers to health care facility G-ASET scores in the top quartile.

## DISCUSSION

We evaluated ASP implementation in 39 HCFs from 5 Latin American countries. We found that HCFs with a higher level of ASP implementation based on G-ASET score showed more desirable AU profiles, such as greater guideline compliance and less unnecessary IV vancomycin use in the ICU. Additionally, we found that lowering multidrug-resistant organism infections may be needed to further reduce AU.

Self-assessment surveys have been used to identify gaps in ASP and IPC program implementation [9, 17, 21]. In our study, education was the domain with the lowest score. This is in agreement with the findings from studies evaluating IPC program implementation globally and in Latin America [9, 21]. Furthermore, recent surveys showed that Latin American health care workers have limited awareness of their local AU, AMR patterns, or hospital-acquired infection rates [22, 23], highlighting the urgent need to strengthen health care worker integration in AS and IPC activities.

Most participating HCFs in our study responded that physicians, pharmacists, and microbiologists contributed to the ASP; however, few individuals had protected time to dedicate to AS (ie, it was a responsibility added to other duties), thereby limiting the scope of the ASP. As an example, most participating HCFs had treatment guidelines, but key information to help clinicians optimize AU was frequently missing from guidelines, such as de-escalation options, duration recommendations, or alternative agents for patients with severe penicillin allergy. Another example of the limited scope of ASPs is the finding that most AS activities reported by HCFs (eg, prior authorization, postprescription review with feedback) were restricted to certain units.

Clinical pharmacists with training in infectious diseases and/or AS have unique and complementary skills to those of physicians and microbiologists and are integral members of ASPs [24]. In this study, pharmacist-driven interventions, such as dose adjustments based on renal function or automatic IV-to-oral conversion, were less commonly reported than physician-led interventions. Furthermore, as compared with HCFs with low G-ASET scores, those with high scores showed a higher proportion of antibiotics prescribed for targeted therapy and a higher proportion of guideline-compliant prescriptions but similar proportions of IV vancomycin undergoing therapeutic drug monitoring. These findings suggest a general need to improve integration of pharmacists in ASPs and physicians’ perceptions about the value of trained pharmacists in ASP. More than half of participating HCFs in our study implemented rapid diagnostic tests for infectious diseases (eg, molecular-based panels for detection of sepsis pathogens); however, few had developed an updated antibiogram or used antibiogram data to update treatment guidelines. Previous research has shown the limited benefit of rapid diagnostic tests for the diagnosis of bloodstream infections without ASP intervention, emphasizing the need for stronger collaboration between microbiology and ASP [25]. Barriers to integrate pharmacists and microbiologists to ASP in Latin America have been recently evaluated and published [6].

Hospitals with higher G-ASET scores had less ICU consumption of anti-MRSA agents than those with lower scores after adjusting for the rate of MRSA infections in the unit. A recent study by our team revealed frequent inappropriate prescribing of IV vancomycin in ICUs and general wards in HCFs in Latin America [10]. We did not see a statistically significant difference in consumption of carbapenems or extensively antibiotic-resistant bacteria antibiotics between high- and low-scoring HCFs, which may be due to a limited sample size. We found that gram-negative multidrug-resistant organism infections rates influenced ICU antibiotic consumption, suggesting that improved infection prevention might be needed for lower AU. Altogether, these data emphasize the need for decision makers at the national and subnational levels to identify levers to incentivize implementation and growth of ASPs and IPC programs in HCFs in Latin America.

There are several limitations to this study. While we included a diverse cohort of HCFs, this was a convenience sample; therefore, the findings might not be generalizable to other HCFs in the region. Self-assessments may overestimate the level of program implementation, as it is human nature to give more desirable responses. Additionally, while the survey utilized for this study was developed by a multidisciplinary team and delivered in the participants’ language (Spanish) following validation by health care workers from Latin America, participants may not have interpreted questions as intended. For example, handshake stewardship and postprescription review with feedback were reported at similar rates by participating HCFs. We are aware that in many Latin American HCFs, especially in medium and small HCFs, AS activities and infectious disease consultation are performed by the same individual; hence, these activities are performed simultaneously and with a different structure than in countries with more resources for ASP. For example, in the United States, handshake stewardship refers to in-person real-time discussion of antibiotic prescriptions by the AS team with the unit team, while postprescription review and feedback are usually performed for selected agents according to prespecified criteria (eg, broad-spectrum activity, cost) at 48 to 72 hours and are typically done by phone, although both these activities may be interpreted as being done when recommendations are discussed during a consult [20, 26]. In-person completion of the G-ASET with guidance from an external reviewer could help mitigate some of these issues related to question interpretation. Despite possible overestimation of the level of ASP implementation with the G-ASET, we were able to validate the tool with AU data from ICUs. This is consistent with a study that demonstrated a positive correlation between higher scores and more desirable AU profiles [17]. As G-ASET assessed ASP structure and activities at the hospital level, it would be helpful for future studies to evaluate the impact of ASPs on AU outside the ICU. We considered ICU infection rates in our regression model, as high infection rates would influence AU; however, our approach to determine a new infection relied on positive microbiologic cultures and clinical interpretation by local teams. While local teams were provided guidance on how to adjudicate cases, we did not assess culture incidence, which could have over- or underestimated infection rates. Finally, the G-ASET does not assess IPC activities. This information could provide insightful information regarding the relationship between AU and AMR.

## CONCLUSIONS

In summary, a multicenter evaluation of ASP implementation in Latin American HCFs based on the G-ASET coupled with ICU longitudinal point prevalence surveys and antibiotic consumption data collected over 12 months showed an association between higher G-ASET scores (ie, level of ASP implementation) and more optimal AU in ICUs. Opportunities to strengthen ASPs in the region based on our evaluation include increasing protected time for health care workers in ASPs; greater integration of microbiologists, clinical pharmacists, and health care workers in ASPs; and improving training opportunities and infection prevention activities.

## Supplementary Material

ofaf364_Supplementary_Data

## Supplementary Data

Supplementary materials are available at *Open Forum Infectious Diseases* online. Consisting of data provided by the authors to benefit the reader, the posted materials are not copyedited and are the sole responsibility of the authors, so questions or comments should be addressed to the corresponding author.

## Notes

***Author contributions.*** V. F., S. E. C., and R. E. Q.: designed the study; collected data, analyzed, and interpreted the data; and wrote the manuscript. T. S. P. and F. C. L.: designed the study, assisted with interpretation of the data, and critically reviewed the manuscript. A. A., B. A., A. B. A., M. F. B., M. D. C. B., M. P. B., M. L. B, A. C., X. C., A. M. C., R. C., W. C., S. M. C., G. C. C., M. I. C. A., L. E., C. Esquivel, C. Ezcurra, J. F., S. F., N. F., C. G. C., M. I. G., C. H. G. Q., J. A. G., L. G., F. G.-T., S. L., D. L., I. L. L., H. M., N. M., D. M. M., M. M., F. M., C. M. P., C. M. I., L. G. M., Y. N., G. N., A. O., B. P., A. P., M. d. P. S., F. P., L. P. A., C. L. R., F. R., L. V. R., G. R., V. E. R., F. R., J. J. R., M. R., M. E. R., G. S., N. Sandoval, N. Staffolani, M. J. T., A. M. U., H. V., M. V., S. V. A. P., H. V.-S., S. V., O. V., E. W., and G. R.-M. contributed to data acquisition, interpretation of results, and review of the manuscript. Y. J. H. analyzed and interpreted data; and drafted the manuscript. All authors have approved the final manuscript.

***Availability of data and materials.*** All data generated for the study are presented in the article and Supplementary material.

***Patient consent statement.*** This study was approved as exempt research by the Johns Hopkins Medicine Institutional Review Board (IRB00299350, IRB00299607).

***Financial support.*** This work was supported by the US Centers for Disease Control and Prevention through a Broad Agency Announcement (contract 75D30121C11953).
