# Supplementary material for: Multicenter Evaluation of Antibiotic Use and Antibiotic Stewardship Programs in Latin American Hospitals
Source: Open Forum Infect Dis. 2025 Jun 25;12(7):ofaf364. doi: 10.1093/ofid/ofaf364 (PMC12272052; doi:10.1093/ofid/ofaf364)
Supplement: ofaf364_Supplementary_Data [file ofaf364_supplementary_data.docx]

# **Supplementary Material**

**“Multicenter Evaluation of Antibiotic Stewardship Programs in Latin American Hospitals: Key Opportunities for Action”**

**Table 1**: National Healthcare Safety Network Adult Antimicrobial Groupings.

**Table 2**: Description of antibiotic use indicators.

**Table 3**: Clinical scenarios of new infection due to multi-drug-resistant organisms (MDROs) in participating ICUs.

**Table 4**: Responses to the self-assessment by the thirty-nine participating healthcare facilities (HCF).

**Table 5**: Annual overall antibiotic consumption (DDD/100 patient-days) for medical-surgical ICUs among the twenty-nine healthcare facilities that collected data.

**Table 6**. Correlation matrix of G-ASET scores and annual antibiotic consumption for medical-surgical intensive care units.

**Table 7**: Aggregate antibiotic use indicators calculated for thirty-seven healthcare facilities that performed point prevalence surveys in an adult medical-surgical ICU during the study period.

**Table 1:** National Healthcare Safety Network (NHSN) Adult Antimicrobial Groupings*

| **Adult Broad spectrum antibacterial agents predominantly used for HAIs** | **Adult broad-spectrum antibacterial agents predominantly used for CAIs** | **Adult antibacterial agents predominantly used for resistant Gram-positive infections** | **Adult narrow-spectrum β-lactam agents** | **Adult antibacterial agents predominantly used for extensively antibiotic-resistant bacteria** |
| --- | --- | --- | --- | --- |
| AMIKACIN (IV only) | CEFACLOR | CEFTAROLINE | AMOXICILLIN | CEFTAZIDIME/AVIBACTAM |
| AZTREONAM (IV only) | CEFDINIR | DALBAVANCIN | AMOXICILLIN/CLAVULANATE | CEFTOLOZANE/TAZOBACTAM |
| CEFEPIME | CEFIXIME | DAPTOMYCIN | AMPICILLIN | COLISTIMETHATE (IV only) |
| CEFTAZIDIME | CEFOTAXIME | LINEZOLID | AMPICILLIN/SULBACTAM | POLYMYXIN B (IV only) |
| DORIPENEM | CEFPODOXIME | ORITAVANCIN | CEFADROXIL | TIGECYCLINE |
| GENTAMICIN (IV only) | CEFPROZIL | QUINUPRISTIN/DALFOPRISTIN | CEFAZOLIN |  |
| IMIPENEM/CILASTATIN | CEFTRIAXONE | TEDIZOLID | CEFOTETAN |  |
| MEROPENEM | CEFUROXIME | TELAVANCIN | CEFOXITIN |  |
| PIPERACILLIN/TAZOBACTAM | CIPROFLOXACIN | VANCOMYCIN (IV only) | CEPHALEXIN |  |
| TOBRAMYCIN (IV only) | ERTAPENEM |  | DICLOXACILLIN |  |
|  | GEMIFLOXACIN |  | NAFCILLIN |  |
|  | LEVOFLOXACIN |  | OXACILLIN |  |
|  | MOXIFLOXACIN |  | PENICILLIN G |  |
|  |  |  | PENICILLIN V |  |

HAI: healthcare-associated infections, CAI: community-acquired infections.

*<https://www.cdc.gov/nhsn/pdfs/pscmanual/11pscaurcurrent.pdf>

**Table 2.** Description of antibiotic use indicators.

| **Indicator** | **Numerator** | **Denominator** |
| --- | --- | --- |
| Directed therapy | Number of antibiotics prescribed as directed therapy (i.e., once culture results area available) | All antibiotics prescribed for therapeutic indication (i.e., not for prophylaxis) |
| De-escalation | Number of antibiotics prescribed as directed therapy that have narrower spectrum than empiric antibiotic | All antibiotics prescribed for directed therapy |
| Anti-MRSA with documented MRSA infection | Number of anti-MRSA agents with documented MRSA infection | Anti-MRSA agents prescribed for targeted therapy |
| Guideline compliance | Number of antibiotics adjudicated as “guideline compliant” | All antibiotics prescribed for empiric or targeted treatment |
| Renal adjustment | Number of antibiotics with appropriate dose adjustment based on renal function | All antibiotics prescribed for empiric or targeted treatment that required renal adjustment |
| IV Vancomycin undergoing therapeutic drug monitoring | Number of IV Vancomycin prescriptions undergoing therapeutic drug monitoring | All IV Vancomycin |

**Table 3.** Clinical scenarios of new infection due to multi-drug resistant organisms (MDROs) in participating ICUs. Infections due to methicillin-resistant *Staphylococcus aureus* (MRSA), extended spectrum-β lactamase (ESBL)-producing Enterobacterales, and carbapenem-resistant (CR) Enterobacterales were included. Cases were determined by local antibiotic stewardship teams based on positive cultures and clinical criteria using guidance provided by the study team. A new infection was one occurring 48 hs after unit admission. The following scenarios illustrate how teams would have counted number of infections.

| **Clinical scenario** | **Number of infections** |
| --- | --- |
| Patient is on mechanical ventilation due to respiratory failure associated with polytrauma. Four days after admission, patient develops ventilator-associated pneumonia due to *K. pneumoniae* ESBL (respiratory culture). Ten days after this episode, the patient develops a catheter-associated urinary tract infection due to a *K. pneumoniae* ESBL (urine culture). | Two |
| Patient is on mechanical ventilation due to respiratory failure associated with polytrauma. Four days after admission, patient develops ventilator-associated pneumonia due to *K. pneumoniae* ESBL. A day later blood cultures are positive for *K. pneumoniae* ESBL. | One |
| Patient is transferred to the unit from another facility for management of shock due to *K. pneumoniae* ESBL infection. A urinary catheter is placed for monitoring of urine output. Six days later, patient develops a catheter-associated urinary tract infection due to a *K. pneumoniae* ESBL (urine culture). | One (infection present on admission is not counted) |
| Patient is admitted to the unit for management of shock due to gastrointestinal bleeding. Three days after admission, patient decompensates. Blood cultures grow *E. coli* ESBL and carbapenem resistant *K. pneumoniae*. | Two |

**Table 4.** Responses to the Global Antibiotic Stewardship Evaluation Tool (G-ASET) by 39 participating healthcare facilities (HCF) in Latin America, overall and by ownership. Country of 39 HCFs included Argentina (n=22, 56.4%), Colombia (n=4, 10.3%), Ecuador (n=4, 10.3%), Guatemala (n=4, 10.3%) and Panama (n=5, 12.8%)

| **Questions included in the self-assessment by domain** | **Overall**  **n=39 (%)** | **For-profit**  **n=17 (%)** | **Non-profit**  **n=22 (%)** | ***P* value** |
| --- | --- | --- | --- | --- |
| **DOMAIN I: LEADERSHIP COMMITMENT & ACCOUNTABILITY** | | |  |  |
| 1. Is AS identified as a priority by HCF management/leadership? |  |  |  | **0.042** |
| Yes | 18 (46.1) | 11 (64.7) | 7 (31.8) |  |
| Partially implemented | 20 (51.2) | 5 (29.4) | 15 (68.1) |  |
| No | 1 (2.5) | 1 (5.8) | - |  |
| 1. Are AS activities included in HCF annual plans with key performance indicators? |  |  |  | **0.049** |
| Yes | 19 (48.7) | 12 (70.5) | 7 (31.8) |  |
| Partially implemented | 14 (35.8) | 4 (23.5) | 10 (45.4) |  |
| No | 6 (15.3) | 1 (5.8) | 5 (22.7) |  |
| 1. Is there a mechanism to regularly monitor and measure the implementation of AS activities? |  |  |  | 0.651 |
| Yes | 20 (51.2) | 9 (52.9) | 11 (50) |  |
| Partially implemented | 12 (30.7) | 6 (35.2) | 6 (27.2) |  |
| No | 7 (17.9) | 2 (11.7) | 5 (22.7) |  |
| 1. Does your HCF have an AS committee that reviews policies, procedures, treatment guidelines, and operational considerations related to AS? |  |  |  | 0.538 |
| Yes | 19 (48.7) | 10 (58.8) | 9 (40.9) |  |
| Partially implemented | 14 (35.8) | 5 (29.4) | 9 (40.9) |  |
| No | 6 (15.3) | 2 (11.7) | 4 (18.1) |  |
| 1. Who are the members of the AS committee at your HCF?  - IPC nurse(s) - Non-IPC nurse(s) - ID trained physician(s) or clinician(s) with experience practicing ID - Intensive care unit physician(s) - Surgeon - General medicine physician(s) - Other physician(s) - ID trained pharmacist(s) or pharmacist with experience practicing ID - Other clinical pharmacist(s) - Other staff pharmacist(s) - Senior HCF leader(s) - Clinical microbiologist(s) - Information technology specialist(s) - Other, please specify: |  |  |  | 0.164 |
| 5 points = ≥7 healthcare professionals selected | 20 (51.2) | 11 (64.7) | 9 (40.9) |  |
| 2.5 points = 1-7 healthcare professionals selected | 16 (41) | 6 (35.2) | 10 (45.4) |  |
| 0 points = none or not applicable | 3 (7.6) |  | 3 (13.6) |  |
| 1. Does the AS committee meet on a regular basis (minimum monthly or quarterly)? |  |  |  | 0.633 |
| Yes | 21 (53.8) | 9 (52.9) | 12 (54.5) |  |
| Partially implemented | 9 (23.07) | 5 (29.4) | 4 (18.1) |  |
| No | 9 (23.07) | 3 (17.6) | 6 (27.2) |  |
| 1. Who are the members of the AS team at your HCF? |  |  |  | 0.482 |
| 5 points = ID trained physician or clinician with experience practicing ID AND ID trained pharmacist(s) OR other clinical pharmacist(s) OR other staff pharmacist(s) (if pharmacists are present at HCF) AND clinical microbiologist (if microbiologist is present at HCF) | 25 (64.1) | 10 (58.8) | 15 (68.1) |  |
| 2.5 points = anything selected that does not meet criteria for 5 points | 13 (33.3) | 7 (41.1) | 6 (27.2) |  |
| 0 points = none or not applicable | 1 (2.5) | - | 1 (4.5) |  |
| 1. Does the AS team meet on a regular basis? |  |  |  |  |
| Yes | 20 (51.2) | 8 (47) | 12 (54.5) | 0.833 |
| Partially implemented | 14 (35.8) | 7 (41.1) | 7 (31.8) |  |
| No | 5 (12.8) | 2 (11.7) | 3 (13.6) |  |
| 1. Does the AS committee or team have authority to make decisions about policies or procedures related to antibiotic use at your HCF? |  |  |  | **0.036** |
| Yes | 21 (53.8) | 13 (76.4) | 8 (36.3) |  |
| Partially implemented | 12 (30.7) | 2 (11.7) | 10 (45.4) |  |
| No | 6 (15.3) | 2 (11.7) | 4 (18.1) |  |
| 1. Which hospital department(s) or healthcare teams does your AS committee or team collaborate with?  - Infection prevention and control - ID - Quality - Pharmacy - Microbiology - Drug and therapeutics committee - HIV/tuberculosis (TB) team - Surgery or operating theater - Other, please specify: - Not applicable |  |  |  | 0.373 |
| 5 points = ≥2 selected | 38 (97.4) | 17 (100) | 21 (95.4) |  |
| 2.5 points = 1 selected |  |  |  |  |
| 0 points = none or not applicable | 1 (2.5) |  | 1 (4.5) |  |
| 1. Does the HCF participate in any external networks (e.g., multicenter studies, research or quality improvement collaboratives, data sharing consortiums) related to AS? |  |  |  | 0.634 |
| Yes | 27 (69.2) | 13 (76.4) | 14 (63.6) |  |
| Partially implemented | 8 (20.5) | 3 (17.6) | 5 (22.7) |  |
| No | 4 (10.2) | 1 (5.8) | 3 (13.6) |  |
| 1. Who is involved in antibiotic formulary/procurement decisions at your HCF?  - ID trained physician(s) or clinician(s) with experience practicing ID - ID trained pharmacist(s) or pharmacist with experience practicing ID - Other clinical pharmacist(s) or other staff pharmacist(s) - Member(s) of AS team - Clinical microbiologist(s) - Other, please specify: - Not applicable |  |  |  | 0.414 |
| 5 points = infectious diseases trained physician or clinician with  experience practicing infectious diseases AND infectious diseases trained pharmacist(s) OR other clinical pharmacist(s) OR other staff pharmacist(s) (if pharmacists are present at HCF) AND clinical microbiologist (if microbiologist is present at HCF) | 12 (30.7) | 4 (23.5) | 8 (36.3) |  |
| 2.5 points = some selected but does not meet criteria for 5 points | 23 (58.9) | 12 (70.5) | 11 (50) |  |
| 0 points = none or not applicable | 4 (10.2) | 1 (5.8) | 3 (13.6) |  |
| 1. Is the evidence related to the safety, efficacy, and cost of new antibiotics evaluated before adding to the formulary at your HCF? |  |  |  | 0.862 |
| Yes | 34 (87.1) | 15 (88.2) | 19 (86.3) |  |
| Partially implemented | 5 (12.8) | 2 (11.7) | 3 (13.6) |  |
| No |  |  |  |  |
| **DOMAIN II: RESOURCES** | | | |  |
| 1. Has the HCF allocated human and financial resources to initiate AS activities? |  |  |  | 0.380 |
| Yes | 6 (15.3) | 4 (23.5) | 2 (9) |  |
| Partially implemented | 13 (33.3) | 6 (35.2) | 7 (31.8) |  |
| No | 20 (51.2) | 7 (41.1) | 13 (59) |  |
| 1. Which of the following are physically present at your HCF? |  |  |  | 0.576 |
| 5 points = infectious diseases trained physician or clinician with experience practicing infectious diseases AND infectious diseases trained pharmacist(s) OR other clinical pharmacist(s) OR other staff pharmacist(s) (if pharmacists are present at HCF) AND clinical microbiologist (if microbiologist is present at HCF) | 26 (66.6) | 11 (64.7) | 15 (68.1) |  |
| 2.5 points = anything selected that does not meet criteria for 5 points | 9 (23.07) | 5 (29.4) | 4 (18.1) |  |
| 0 points = none or not applicable No | 4 (10.2) | 1 (5.8) | 3 (13.6) |  |
| 1. Does the AS team have an office or physical space to perform AS activities? |  |  |  | 0.212 |
| Yes | 17 (43.5) | 8 (47) | 9 (40.9) |  |
| Partially implemented | 7 (17.9) | 1 (5.8) | 6 (27.2) |  |
| No | 15 (38.4) | 8 (47) | 7 (31.8) |  |
| 1. Does the AS team have the basic equipment (e.g., telephone, computer) to perform AS activities? |  |  |  | 0.383 |
| Yes | 21 (53.8) | 9 (52.9) | 12 (54.5) |  |
| Partially implemented | 10 (25.6) | 3 (17.6) | 7 (31.8) |  |
| No | 8 (20.5) | 5 (29.4) | 3 (13.6) |  |
| 1. Does your HCF have information and decision support systems in place to support AS activities (e.g., review and optimization of antibiotic prescriptions, pre-authorization)? |  |  |  | 0.061 |
| Yes | 30 (76.9) | 16 (94.1) | 14 (63.6) |  |
| Partially implemented | 4 (10.2) | 1 (5.8) | 3 (13.6) |  |
| No | 5 (12.8) | - | 5 (22.7) |  |
| 1. Which of the following can the AS team access?  - Electronic medical record - Antibiotics purchased - Antibiotics dispensed - Antibiotic administration records - Syndromic antibiogram (e.g., antibiogram with urine cultures) - Cumulative antibiogram - Not applicable |  |  |  | 0.373 |
| 5 points = ≥2 selected | 38 (97.4) | 17 (100) | 21 (95.4) |  |
| 2.5 points = 1 selected | - | - | - |  |
| 0 points = none or not applicable | 1 (2.5) | - | 1 (4.5) |  |
| 1. Which data are available electronically at your HCF?  - Antibiotic consumption - Antibiotic use - Antibiotic resistance - Antibiotic cost - Administrative data (e.g., patient days, discharges) - Other, please specify: |  |  |  | 0.179 |
| 5 points = ≥2 selected | 35 (89.7) | 17 (100) | 18 (81.8) |  |
| 2.5 points = 1 selected | 3 (7.6) |  | 3 (13.6) |  |
| 0 points = none or not applicable | 1 (2.5) |  | 1 (4.5) |  |
| 1. Does the AS team have access to updated evidence in the form of peer-reviewed scientific literature (e.g., published research)? |  |  |  | 0.608 |
| Yes | 28 (72) | 11 (64.7) | 17 (77.2) |  |
| Partially implemented | 8 (20.5) | 4 (23.5) | 4 (18.1) |  |
| No | 3 (7.6) | 2 (11.7) | 1 (4.5) |  |
| 1. Does the HCF have access to laboratory and imaging services (on-site or off-site) that can be used to support AS interventions? |  |  |  | 0.443 |
| Yes | 37 (94.8) | 17 (100) | 20 (90.9) |  |
| Partially implemented | 1 (2.5) | - | 1 (4.5) |  |
| No | 1 (2.5) | - | 1 (4.5) |  |
| 1. Is the clinical microbiology laboratory used by your HCF (on-site or off-site) open 24 hours per day to receive, process, and report microbiologic specimens? |  |  |  | 0.238 |
| Yes | 20 (51.2) | 11 (64.7) | 9 (40.9) |  |
| Partially implemented | 15 (38.4) | 4 (23.5) | 11 (50) |  |
| No | 4 (10.2) | 2 (11.7) | 2 (9) |  |
| 1. Is the clinical microbiology laboratory used by your HCF (on-site or off-site) accredited? |  |  |  | 0.329 |
| Yes | 33 (84.6) | 16 (94.1) | 17 (77.2) |  |
| Partially implemented | 5 (12.8) | 1 (5.8) | 4 (18.1) |  |
| No | 1 (2.5) | - | 1 (4.5) |  |
| 1. Does the clinical microbiology laboratory used by your HCF (on-site or off-site) have a quality management system? |  |  |  | 0.493 |
| Yes | 31 (79.4) | 15 (88.2) | 16 (72.7) |  |
| Partially implemented | 4 (10.2) | 1 (5.8) | 3 (13.6) |  |
| No | 4 (10.2) | 1 (5.8) | 3 (13.6) |  |
| 1. Does the clinical microbiology laboratory used by your HCF (on-site or off-site) have an electronic laboratory information system? |  |  |  | 0.655 |
| Yes | 33 (84.6) | 15 (88.2) | 18 (81.8) |  |
| Partially implemented | 5 (12.8) | 2 (11.7) | 3 (13.6) |  |
| No | 1 (2.5) |  | 1 (245) |  |
| **DOMAIN III: EDUCATION & TRAINING** | | | |  |
| 1. Does the HCF provide training on AS to staff during induction training? |  |  |  | 0.186 |
| Yes | 14 (35.8) | 8 (47) | 6 (27.2) |  |
| Partially implemented | 15 (38.4) | 7 (41.1) | 8 (36.3) |  |
| No | 10 (25.6) | 2 (11.7) | 8 (36.3) |  |
| 1. Does the HCF offer continuous in-service training or continuous professional development on AS and IPC to staff? |  |  |  | 0.432 |
| Yes | 11 (28.2) | 6 (35.2) | 5 (22.7) |  |
| Partially implemented | 20 (51.2) | 9 (52.9) | 11 (50) |  |
| No | 8 (20.5) | 2 (11.7) | 6 (27.2) |  |
| 1. Does your HCF provide training on AS to students or trainees rotating at your HCF? |  |  |  | 0.296 |
| Yes | 15 (38.4) | 7 (41.1) | 8 (36.3) |  |
| Partially implemented | 4 (10.2) | 4 (23.5) | 10 (45.4) |  |
| No | 10 (25.6) | 6 (35.2) | 4 (18.1) |  |
| 1. Does the HCF provide training for the AS team on AS/IPC? |  |  |  | 0.345 |
| Yes | 18 (46.1) | 9 (52.9) | 9 (40.9) |  |
| Partially implemented | 15 (38.4) | 7 (41.1) | 8 (36.3) |  |
| No | 6 (15.3) | 1 (5.8) | 5 (22.7) |  |
| 1. Does your HCF provide patients and/or families with education about antibiotics? |  |  |  |  |
| Yes | 8 (20.5) | 4 (23.5) | 4 (18.1) | **0.012** |
| Partially implemented | 12 (30.7) | 9 (52.9) | 3 (13.6) |  |
| No | 19 (48.7) | 4 (23.5) | 15 (68.1) |  |
| **DOMAIN IV: ANTIMICROBIAL STEWARDSHIP ACTIONS** | | | |  |
| 1. Which of the following treatment guidelines exist at your HCF?  - Urinary tract infection - Community-acquired pneumonia - Ventilator-associated pneumonia - Sepsis - Skin and soft tissue infection - Surgical site infection - Central line-associated bloodstream infection - Surgical prophylaxis - Intra-abdominal infection - Febrile neutropenia - Management of multidrug-resistant organisms - Bacterial meningitis - Infective endocarditis - Other, please specify: - Not applicable (no treatment guidelines exist at my HCF) |  |  |  | 0.851 |
| 5 points = ≥4 selected | 37 (94.8) | 16 (94.1) | 21 (95.4) |  |
| 2.5 points = 1-3 selected | 2 (5.1) | 1 (5.8) | 1 (4.5) |  |
| 0 points = none or not applicable |  |  |  |  |
| 1. Which of the following is/are included in treatment guidelines at your HCF? |  |  |  | 0.981 |
| 5 points = first-line antibiotic agent, dose, duration, alternative antibiotic agents selected | 16 (41) | 7 (41.1) | 9 (40.9) |  |
| 2.5 points = anything selected that does not meet criteria for 5 points | 21 (53.8) | 9 (52.9) | 12 (54.5) |  |
| 0 points = none or not applicable | 2 (5.1) | 1 (5.8) | 1 (4.5) |  |
| 1. Are the guidelines reviewed and updated periodically based on availability of new evidence, with changes communicated to prescribers? |  |  |  | 0.117 |
| Yes | 22 (56.4) | 12 (70.5) | 10 (45.4) |  |
| Partially implemented | 17 (43.5) | 5 (29.4) | 12 (54.5) |  |
| No |  |  |  |  |
| 1. Does the AS team review the HCF antibiogram on a regular basis to modify treatment guidelines? |  |  |  | 0.963 |
| Yes | 22 (56.4) | 10 (58.8) | 12 (54.5) |  |
| Partially implemented | 12 (30.7) | 5 (29.4) | 7 (31.8) |  |
| No | 5 (12.8) | 2 (11.7) | 3 (13.6) |  |
| 1. Is this activity routinely conducted at your HCF?  - Antibiotic automatic stops (e.g., surgical prophylaxis) - Antibiotic "time outs" (defined, regular prompts to the clinician to re-evaluate antibiotic choices) - Antibiotic reminders (alerts at the time of prescribing) - Intravenous to oral antibiotic formulation conversion - Prospective audit and feedback of specified antibiotics - Prior authorization of specified antibiotics - Restricted use of antibiotics based on formulary approval for prespecified conditions or populations - Individual audit and feedback of performance to prescribers - In-person AS clinical rounds - Assessment and clarification of documented antibiotic allergies - Review of outpatient parenteral antibiotic therapy prior to discharge - Alerts to prescribers about potentially duplicative antibiotic coverage (e.g., double anti-anaerobic coverage) - Alerts to prescribers about drug-drug interactions - Dose optimization based on pharmacokinetic and pharmacodynamic parameters for treatment of organisms with reduced antibiotic susceptibility - Management of antibiotic shortages/stockouts - Pharmacist-assisted or clinical pharmacologist-assisted dosing of antibiotics in patients with renal or liver dysfunction - Therapeutic drug monitoring of antibiotics with narrow therapeutic index (e.g., vancomycin, aminoglycosides) - Awareness campaigns on responsible use of antibiotics |  |  |  | 0.194 |
| 5 points = ≥9 "yes" selected | 31 (79.4) | 12 (70.5) | 19 (86.3) |  |
| 2.5 points = 1-8 "yes" selected | 7 (17.9) | 5 (29.4) | 2 (9) |  |
| 0 points = none selected | 1 (2.5) |  | 1 (4.5) |  |
| 1. If yes, is the activity performed facility-wide? |  |  |  |  |

| Antibiotic automatic stops (e.g., surgical prophylaxis) |  |  |  |  |
| --- | --- | --- | --- | --- |
| - Yes | 12 (30.8) | 7 (41.2) | 5 (22.7) |  |
| - No | 8 (20.5) | 3 (17.6) | 5 (22.7) |  |
| - No answer provided | 19 (48.7) | 7 (41.2) | 12 (54.5) |  |
| Antibiotic "time outs" (defined, regular prompts to the clinician to re-evaluate antibiotic choices) |  |  |  |  |
| - Yes | 13 (33.3) | 7 (41.2) | 6 (27.3) |  |
| - No | 8 (20.5) | 3 (17.6) | 5 (22.7) |  |
| - No answer provided | 18 (46.2) | 7 (41.2) | 11 (50) |  |
| Antibiotic reminders (alerts at the time of prescribing) |  |  |  |  |
| - Yes | 12 (30.8) | 6 (35.3) | 6 (27.3) |  |
| - No | 7 (17.9) | 4 (23.5) | 3 (13.6) |  |
| - No answer provided | 20 (51.3) | 7 (41.2) | 13 (59.1) |  |
| Intravenous to oral antibiotic formulation conversion |  |  |  |  |
| - Yes | 4 (10.3) | 3 (17.6) | 1 (4.5) |  |
| - No | 12 (30.8) | 5 (29.4) | 7 (31.8) |  |
| - No answer provided | 23 (59) | 9 (52.9) | 14 (63.6) |  |
| Prospective audit and feedback of specified antibiotics |  |  |  |  |
| - Yes | 12 (30.8) | 6 (35.3) | 6 (27.3) |  |
| - No | 10 (25.6) | 3 (17.6) | 7 (31.8) |  |
| - No answer provided | 17 (43.6) | 8 (47.1) | 9 (40.9) |  |
| Prior authorization of specified antibiotics |  |  |  |  |
| - Yes | 18 (46.2) | 11 (64.7) | 7 (31.8) |  |
| - No | 8 (20.5) | 3 (17.6) | 5 (22.7) |  |
| - No answer provided | 13 (33.3) | 3 (17.6) | 10 (45.5) |  |
| Restricted use of antibiotics based on formulary approval for prespecified conditions or populations |  |  |  |  |
| - Yes | 13 (33.3) | 7 (41.2) | 6 (27.3) |  |
| - No | 8 (20.5) | 2 (11.8) | 6 (27.3) |  |
| - No answer provided | 18 (46.2) | 8 (47.1) | 10 (45.5) |  |
| Individual audit and feedback of performance to prescribers |  |  |  |  |
| - Yes | 16 (41) | 11 (64.7) | 5 (22.7) |  |
| - No | 10 (25.6) | 3 (17.6) | 7 (31.8) |  |
| - No answer provided | 13 (33.3) | 3 (17.6) | 10 (45.5) |  |
| In-person antibiotic stewardship clinical rounds |  |  |  |  |
| - Yes | 12 (30.8) | 6 (35.3) | 6 (27.3) |  |
| - No | 14 (35.9) | 7 (41.2) | 7 (31.8) |  |
| - No answer provided | 13 (33.3) | 4 (23.5) | 9 (40.9) |  |
| Assessment and clarification of documented antibiotic allergies |  |  |  |  |
| - Yes | 17 (43.6) | 11 (64.7) | 6 (27.3) |  |
| - No | 6 (15.4) | 2 (11.8) | 4 (18.2) |  |
| - No answer provided | 16 (41) | 4 (23.5) | 12 (54.5) |  |
| Review of outpatient parenteral antibiotic therapy prior to discharge |  |  |  |  |
| - Yes | 6 (15.4) | 6 (35.3) | - |  |
| - No | 8 (20.5) | 2 (11.8) | 6 (27.3) |  |
| - No answer provided | 24 (61.5) | 9 (52.9) | 15 (68.2) |  |
| Alerts to prescribers about potentially duplicative antibiotic coverage (e.g., double anti-anaerobic coverage) |  |  |  |  |
| - Yes | 9 (23.1) | 5 (29.4) | 4 (18.2) |  |
| - No | 9 (23.1) | 4 (23.5) | 5 (22.7) |  |
| - No answer provided | 21 (53.8) | 8 (47.1) | 13 (59.1) |  |
| Alerts to prescribers about drug-drug interactions |  |  |  |  |
| - Yes | 11 (28.2) | 8 (47.1) | 3 (13.6) |  |
| - No | 9 (23.1) | 3 (17.6) | 6 (27.3) |  |
| - No answer provided | 19 (48.7) | 6 (35.3) | 13 (59.1) |  |
| Dose optimization based on pharmacokinetic and pharmacodynamic parameters for treatment of organisms with reduced antibiotic susceptibility |  |  |  |  |
| - Yes | 10 (25.6) | 5 (29.4) | 5 (22.7) |  |
| - No | 14 (35.9) | 8 (47.1) | 6 (27.3) |  |
| - No answer provided | 15 (38.5) | 4 (23.5) | 11 (50) |  |
| Management of antibiotic shortages/stockouts |  |  |  |  |
| - Yes | 16 (41) | 7 (41.2) | 9 (40.9) |  |
| - No | 7 (17.9) | 3 (17.6) | 4 (18.2) |  |
| - No answer provided | 16 (41) | 7 (41.2) | 9 (40.9) |  |
| Pharmacist-assisted or clinical pharmacologist-assisted dosing of antibiotics in patients with renal or liver dysfunction |  |  |  |  |
| - Yes | 10 (25.6) | 7 (41.2) | 3 (13.6) |  |
| - No | 9 (23.1) | 3 (17.6) | 6 (27.3) |  |
| - No answer provided | 20 (51.3) | 7 (41.2) | 13 (59.1) |  |
| Therapeutic drug monitoring of antibiotics with narrow therapeutic index (e.g., vancomycin, aminoglycosides) |  |  |  |  |
| - Yes | 18 (46.2) | 11 (64.7) | 7 (31.8) |  |
| - No | 8 (20.5) | 4 (23.5) | 4 (18.2) |  |
| - No answer provided | 13 (33.3) | 2 (11.8) | 11 (50) |  |
| Awareness campaigns on responsible use of antibiotics |  |  |  |  |
| - Yes | 15 (38.5) | 8 (47.1) | 7 (31.8) |  |
| - No | 10 (25.6) | 6 (35.3) | 4 (18.2) |  |
| - No answer provided | 14 (35.9) | 3 (17.6) | 11 (50) |  |

| 1. Are there standardized operating procedures for specific AS activities (e.g., audit and feedback, guideline development, testing protocols) at your HCF? |  |  |  | 0.181 |
| --- | --- | --- | --- | --- |
| Yes | 13 (33.3) | 8 (47) | 5 (22.7) |  |
| Partially implemented | 17 (43.5) | 7 (41.1) | 10 (45.4) |  |
| No | 9 (23.1) | 2 (11.7) | 7 (31.8) |  |
| 1. Does the activity report produced by the AS committee or team include the following?  - Current AS resources and activity - Performance against process and outcome indicators for antibiotic use - Antibiotic appropriateness - Antibiotic resistance - Key areas of improvement - Areas for further improvement or priority - Areas in which guidance or support from executive and governance units is needed |  |  |  | 0.247 |
| 5 points = ≥2 selected | 34 (87.1) | 14 (83.3) | 20 (90.9) |  |
| 2.5 points = 1 selected | 2 (5.1) | 2 (11.7) |  |  |
| 0 points = none or not applicable | 3 (7.6) | 1 (5.8) | 2 (9) |  |
| 1. Who is the AS activity report disseminated to?  - HCF management - Other HCF team members - National authorities (e.g., ministry of health) |  |  |  | 0.771 |
| 5 points = ≥2 selected | 26 (66.6) | 12 (70.5) | 14 (63.6) |  |
| 2.5 points = 1 selected | 9 (23.07) | 3 (17.6) | 6 (27.2) |  |
| 0 points = none or not applicable | 4 (10.2) | 2 (11.7) | 2 (9) |  |
| 1. Does the HCF have a formulary/ list of approved antibiotics for use based on the national formulary? |  |  |  | 0.516 |
| Yes | 28 (71.7) | 13 (76.4) | 15 (68.1) |  |
| Partially implemented | 5 (12.8) | 1 (5.8) | 4(18.1) |  |
| No | 6 (15.3) | 3 (17.6) | 3 (13.6) |  |
| 1. Does the HCF formulary specify lists of restricted antibiotics that require approval by a designated team or person (pre-authorization)? |  |  |  | 0.808 |
| Yes | 26 (66.6) | 12 (70.3) | 14 (63.6) |  |
| Partially implemented | 9 (23.07) | 4 (23.5) | 5 (22.7) |  |
| No | 3 (7.6) | 1 (5.8) | 2 (9) |  |
| 1. Does the approval of restricted antibiotics take place throughout the workday? |  |  |  | 0.358 |
| Yes | 25 (64.1) | 13 (76.4) | 12 (54.5) |  |
| Partially implemented | 9 (23.07) | 2 (11.7) | 7 (31.8) |  |
| No | 4 (10.2) | 2 (11.7) | 2 (9) |  |
| 1. Does the HCF communicate modifications to the antibiotic formulary to prescribers? |  |  |  | 0.632 |
| Yes | 21 (53.8) | 10 (58.8) | 11 (50) |  |
| Partially implemented | 7 (17.9) | 2 (11.7) | 5 (22.7) |  |
| No | 10 (25.6) | 5 (29.4) | 5 (22.7) |  |
| 1. Do nurses at your HCF do any of the following AS activities?  - Collect urine and/or respiratory cultures based on appropriate criteria - Initiate discussions about converting from intravenous to oral formulation - Initiate antibiotic "time outs" - Antibiotic allergy assessment |  |  |  | 0.092 |
| 5 points = ≥2 selected | 21 (53.8) | 12 (70.3) | 9 (40.9) |  |
| 2.5 points = 1 selected | 10 (25.6) | 4 (23.5) | 6 (27.2) |  |
| 0 points = none or not applicable | 8 (20.5) | 1 (5.8) | 7 (31.8) |  |
| 1. Does your HCF have a policy that requires prescribers to document antibiotic dose, duration, and indication in the medical record? |  |  |  | 0.178 |
| Yes | 27 (69.2) | 14 (82.3) | 13 (59) |  |
| Partially implemented | 9 (23.07) | 3 (17.6) | 6 (27.2) |  |
| No | 3 (7.6) | - | 3 (13.6) |  |
| 1. Does the clinical microbiology laboratory used by your HCF (on-site or off-site) utilize rapid diagnostic testing to facilitate early antibiotic adjustments? |  |  |  | 0.127 |
| Yes | 23 (58.9) | 12 (70.5) | 11 (50) |  |
| Partially implemented | 11 (28.2) | 2 (11.7) | 9 (40.9) |  |
| No | 5 (12.8) | 3 (17.6) | 2 (9) |  |
| 1. Does the clinical microbiology laboratory used by your HCF (on-site or off-site) have technology to identify the most relevant resistance mechanisms (e.g., extended spectrum beta-lactamases, carbapenemases)? |  |  |  | - |
| Yes | 39 (100) | 17 (100) | 22 (100) |  |
| Partially implemented |  |  |  |  |
| No |  |  |  |  |
| 1. Does the clinical microbiology laboratory used by your HCF (on-site or off-site) provide culture and susceptibility results to prescribers in a timely manner (e.g., within 72 hours)? |  |  |  | 0.063 |
| Yes | 35 (89.7) | 17 (100) | 18 (81.8) |  |
| Partially implemented | 4 (10.2) | - | 4 (18.1) |  |
| No |  |  |  |  |
| 1. Does the clinical microbiology laboratory used by your HCF (on-site or off-site) utilize selective or cascading antibiotic susceptibility testing reporting (e.g., not reporting an antibiotic that would not be appropriate for the source, not reporting a broad-spectrum antibiotic when a narrower spectrum is available)? |  |  |  | 0.350 |
| Yes | 25 (64.1) | 13 (76.4) | 12 (54.5) |  |
| Partially implemented | 6 (15.3) | 2 (11.7) | 4 (18.1) |  |
| No | 8 (20.5) | 2 (11.7) | 6 (27.2) |  |
| 1. Does the clinical microbiology laboratory used by your HCF (on-site or off-site) put comments in microbiology reports to improve antibiotic prescribing? |  |  |  | 0.108 |
| Yes | 25 (64.1) | 14 (82.3) | 11 (50) |  |
| Partially implemented | 7 (17.9) | 2 (11.7) | 6 (27.2) |  |
| No | 6 (15.3) | 1 (5.8) | 5 (22.7) |  |
| 1. Does the AS team communicate the emergence of new resistance mechanisms to prescribers? |  |  |  | 0.907 |
| Yes | 27 (69.2) | 12 (70.5) | 15 (68.1) |  |
| Partially implemented | 8 (20.5) | 3 (17.6) | 5 (22.7) |  |
| No | 4 (10.2) | 2 (11.7) | 2 (9) |  |
| 1. Has the AS team conducted an analysis of the barriers, challenges and opportunities for AS implementation at your HCF? |  |  |  | 0.459 |
| Yes | 17 (43.5) | 7 (41.1) | 10 (45.4) |  |
| Partially implemented | 14 (35.8) | 5 (29.4) | 9 (40.9) |  |
| No | 8 (20.5) | 5 (29.4) | 3 (13.6) |  |
| **DOMAIN V: ANTIBIOTIC USE TRACKING, MONITORING & REPORTING** | | | |  |
| 1. Are regular prescription audits, point prevalence surveys to assess the appropriateness of antibiotic prescribing undertaken at the facility by the AS committee or relevant team? |  |  |  | 0.933 |
| Yes | 24 (61.5) | 10 (58.8) | 14 (63.6) |  |
| Partially implemented | 9 (23.07) | 4 (23.5) | 5 (22.7) |  |
| No | 6 (15.3) | 3 (17.6) | 3 (13.6) |  |
| 1. Does the HCF regularly monitor the quantity and types of antibiotic use (purchased/prescribed/dispensed)? |  |  |  | 0.931 |
| Yes | 22 (56.4) | 10 (58.8) | 12 (54.5) |  |
| Partially implemented | 15 (38.4) | 6 (35.2) | 9 (40.9) |  |
| No | 2 (5.1) | 1 (5.8) | 1 (4.5) |  |
| 1. What metric does the AS team use to measure antibiotic use or   consumption at your HCF? (Select all that apply):   - Days of therapy - Defined daily doses - Other, please specify: |  |  |  | 0.851 |
| 5 points = ≥1 selected | 37 (94.8) | 16 (94.1) | 21 (95.4) |  |
| 0 points = none or not applicable | 2 (5.1) | 1 (5.8) | 1 (4.5) |  |
| 1. Does the AS team develop action plans in response to problems identified related to optimization of antibiotic use (e.g., increase in the consumption of broad-spectrum antibiotics)? |  |  |  | 0.080 |
| Yes | 21 (53.8) | 11 (64.7) | 10 (45.4) |  |
| Partially implemented | 14 (35.8) | 3 (17.6) | 11 (50) |  |
| No | 4 (10.2) | 3 (17.6) | 1 (4.5) |  |
| 1. Does the AS team monitor compliance with at least one specific AS activity (e.g. compliance with treatment guidelines) at the HCF? |  |  |  | 0.687 |
| Yes | 21 (53.8) | 10 (58.8) | 11 (50) |  |
| Partially implemented | 12 (30.7) | 4 (23.5) | 8 (36.3) |  |
| No | 6 (15.3) | 3 (29.4) | 3 (13.6) |  |
| 1. Does the AS committee or team implement strategies (e.g., presentation to healthcare workers on implementation of new ID treatment guideline) to increase compliance with prioritized AS activities? |  |  |  | 0.581 |
| Yes | 17 (43.5) | 8 (47) | 9 (40.9) |  |
| Partially implemented | 19 (48.7) | 7 (41.1) | 12 (54.5) |  |
| No | 3 (7.6) | 2 (11.7) | 1 (4.5) |  |
| 1. Which of the following metrics are monitored by the AS team to assess the impact of AS activities?:  - Antibiotic use or consumption - Antibiotic appropriateness (agent, dose, duration) - Time to appropriate antibiotic therapy - Cost-savings - In-hospital mortality - Length of stay - Clostridioides difficile infection rates - Rehospitalization - Antibiotic-related adverse events - Antibiotic-related near misses (e.g., an error that could have led to an adverse event but did not result in clinical harm) - Other, please specify: |  |  |  | 0.373 |
| 5 points = ≥2 selected | 38 (97.4) | 17 (100) | 21 (95.4) |  |
| 2.5 points = 1 selected | - | - | - |  |
| 0 points = none or not applicable | 1 (2.5) | - | 1 (4.5) |  |
| 1. Which data can be stratified by hospital unit/ward at your HCF?  - Antibiotic consumption - Antibiotic use - Antibiotic resistance - Antibiotic cost - Administrative data (e.g., patient days, discharges) - Other, please specify: |  |  |  | 0.249 |
| 5 points = ≥2 selected | 38 (97.4) | 16 (94.1) | 22 (100) |  |
| 2.5 points = 1 selected | 1 (2.5) | 1 (5.8) | - |  |
| 0 points = none or not applicable | - |  |  |  |
| 1. Does the HCF regularly monitor shortages/stockouts of essential antimicrobials? |  |  |  | 0.301 |
| Yes | 28 (71.7) | 14 (82.3) | 14 (63.6) |  |
| Partially implemented | 9 (23.07) | 3 (17.6) | 6 (27.2) |  |
| No | 2 (5.1) |  | 2 (9) |  |
| 1. Does your HCF monitor shortages/stockouts of laboratory supplies (e.g., reagents, plates)? |  |  |  | 0.725 |
| Yes | 33 (84.6) | 15 (88.2) | 18 (81.8) |  |
| Partially implemented | 4 (10.2) | 1 (5.8) | 3 (13.6) |  |
| No | 2 (5.1) | 1 (5.8) | 1 (4.5) |  |
| 1. If there is a concern about substandard quality of antibiotics (e.g., falsified antibiotics) and diagnostics, is there a mechanism to report this at the HCF? |  |  |  | 0.184 |
| Yes | 20 (51.2) | 11 (64.7) | 9 (40.9) |  |
| Partially implemented | 6 (15.3) | 3 (17.6) | 3 (13.6) |  |
| No | 13 (33.3) | 3 (17.6) | 10 (45.4) |  |
| 1. Does the AS committee or team regularly monitor and report antibiotic susceptibility and resistance rates for a range of key indicator bacteria? |  |  |  | 0.667 |
| Yes | 28 (71.7) | 12 (70.5) | 16 (72.7) |  |
| Partially implemented | 6 (15.3) | 2 (11.7) | 4 (18.1) |  |
| No | 5 (12.8) | 3 (17.6) | 2 (9) |  |
| 1. Does the AS team communicate findings from audits/reviews of the quality/appropriateness of antibiotic use to prescribers along with specific action points? |  |  |  | 0.710 |
| Yes | 12 (30.7) | 6 (35.2) | 6 (27.2) |  |
| Partially implemented | 19 (48.7) | 7 (41.1) | 12 (54.5) |  |
| No | 8 (20.5) | 4 (23.5) | 4 (18.1) |  |
| 1. Does the AS team report metrics used to assess the impact of AS activities to leadership at your HCF? |  |  |  | 0.354 |
| Yes | 16 (41) | 8 (47) | 8 (36.3) |  |
| Partially implemented | 14 (35.8) | 4 (23.5) | 10 (45.4) |  |
| No | 9 (23.1) | 5 (29.4) | 4 (18.1) |  |
| 1. Does the HCF develop an aggregate antibiogram and regularly update it? |  |  |  | 0.524 |
| Yes | 20 (51.2) | 9 (52.9) | 11 (50) |  |
| Partially implemented | 10 (25.6) | 3 (17.6) | 7 (31.8) |  |
| No | 9 (23.1) | 5 (29.4) | 4 (18.1) |  |

Antibiotic Stewardship: AS, Infectious Diseases: ID, HCF: healthcare facility

**Table 5.** Annual overall antibiotic consumption (DDD/100 patient-days) for medical-surgical ICUs among the 29 healthcare facilities that collected data. Study period, March 2022-February 2023.

|  | **Median overall antibiotic consumption** | **IQR** | **Range** | ***P* value** |
| --- | --- | --- | --- | --- |
| Overall, n=29 | 114 | 94 - 144 | 43 - 223 |  |
| Ownership |  |  |  |  |
| For-profit, n=12 | 96 | 88 - 128 | 43 - 161 | 0.063 |
| Not- profit, n=17 | 123 | 108 - 146 | 79 - 223 |  |
| Bed size |  |  |  |  |
| <= 200, n=4 | 105 | 94 - 135 | 79 - 223 | 0.663 |
| 201+, n=15 | 121 | 93 - 146 | 43 - 212 |  |
| Country |  |  |  |  |
| Argentina, =21 | 108 | 90 - 135 | 43 - 164 | 0.421 |
| Ecuador, n=3 | 114 | 94 - 152 | 94 - 152 |  |
| Guatemala, n=1 | 123 | 123 - 123 | 123 - 123 |  |
| Panama, n=4 | 166 | 109 - 217 | 98 - 223 |  |

**Table 6**. Correlation matrix of G-ASET scores and annual antibiotic consumption for medical-surgical intensive care units (n=29). Spearman's correlation coefficients presented.

|  | **Overall score** | **Domain 1 score** | **Domain 2 score** | **Domain 3 score** | **Domain 4 score** | **Domain 5 score** |
| --- | --- | --- | --- | --- | --- | --- |
| **Antibiotic consumption, DDD/100PD** | 1 |  |  |  |  |  |
| **Self-assessment: Overall** | 0.17 | 1 |  |  |  |  |
| **Self-assessment domain 1: Leadership Commitment & Accountability** | 0.03 | 0.81* | 1 |  |  |  |
| **Self-assessment domain 2: Resources** | 0.06 | 0.48* | 0.51* | 1 |  |  |
| **Self-assessment domain 3: Education & Training** | 0.07 | 0.68* | 0.44* | 0.20 | 1 |  |
| **Self-assessment domain 4: AS Actions** | 0.12 | 0.83* | 0.56* | 0.17 | 0.57* | 1 |
| **Self-assessment domain 5: AU Tracking, Monitoring, & Reporting** | 0.22 | 0.78* | 0.55* | 0.23 | 0.51* | 0.56* |

**P* value < 0.05. AS: antibiotic stewardship; AU: antibiotic use.

**Table 7**. Aggregate antibiotic use indicators calculated for thirty-six healthcare facilities that performed point prevalence surveys in an adult medical-surgical ICU during the study period (March 2022-February 2023).

|  | **Median (%)** | **IQR (%)** | **Range (%)** |
| --- | --- | --- | --- |
| Directed therapy | 36.0 | 23.0 - 48.1 | 5.4 - 81.7 |
| De-escalation | 3.4 | 1.6 - 6.0 | 0 - 48.6 |
| Renal adjustment | 96.6 | 86.2 - 100 | 31.8 - 100 |
| Guideline compliance | 94.8 | 80.0 - 98.4 | 9.1 - 100 |
| Anti-MRSA antibiotics prescribed for targeted therapy with documented MRSA infection | 51.9 | 37.2 - 61.4 | 16.7 - 100 |
| Therapeutic drug monitoring of IV vancomycin | 57.1 | 33.3 - 83.3 | 0.0 - 100 |

*Combination in empiric treatment refers to empiric regimens containing IV Vancomycin plus a broad-spectrum agent including carbapenems, 4th or 5th gen Cephalosporin, Piperacillin -tazobactam or a last generation BL/BLI.
